# Redox signalling and mitochondrial stress responses; lessons from inborn errors of metabolism

**DOI:** 10.1007/s10545-015-9861-5

**Published:** 2015-05-30

**Authors:** Rikke K. J. Olsen, Nanna Cornelius, Niels Gregersen

**Affiliations:** Research Unit for Molecular Medicine, Aarhus University Hospital, Palle Juul-Jensens Boulevard 99, 8200 Aarhus N, Denmark; Department of Clinical Medicine, Aarhus University, Palle Juul-Jensens Boulevard 99, 8200 Aarhus N, Denmark; Applied Human Molecular Genetics, Kennedy Center, Copenhagen University Hospital, Rigshospitalet, 2600 Glostrup, Denmark

## Abstract

Mitochondria play a key role in overall cell physiology and health by integrating cellular metabolism with cellular defense and repair mechanisms in response to physiological or environmental changes or stresses. In fact, dysregulation of mitochondrial stress responses and its consequences in the form of oxidative stress, has been linked to a wide variety of diseases including inborn errors of metabolism. In this review we will summarize how the functional state of mitochondria — and especially the concentration of reactive oxygen species (ROS), produced in connection with the respiratory chain — regulates cellular stress responses by redox regulation of nuclear gene networks involved in repair systems to maintain cellular homeostasis and health. Based on our own and other’s studies we re-introduce the ROS triangle model and discuss how inborn errors of mitochondrial metabolism, by production of pathological amounts of ROS, may cause disturbed redox signalling and induce chronic cell stress with non-resolving or compromised cell repair responses and increased susceptibility to cell stress induced cell death. We suggest that this model may have important implications for those inborn errors of metabolism, where mitochondrial dysfunction plays a major role, as it allows the explanation of oxidative stress, metabolic reprogramming and altered signalling growth pathways that have been reported in many of the diseases. It is our hope that the model may facilitate novel ideas and directions that can be tested experimentally and used in the design of future new approaches for pre-symptomatic diagnosis and prognosis and perhaps more effective treatments of inborn errors of metabolism.

## Introduction

The traditional view of inborn errors of metabolism (IEM) is genetic defects in metabolic enzymes or transporters, which result in accumulation of toxic substrates and/or a compromised ability to synthesize essential compounds such as adenosine triphosphate (ATP). However, although these features of IEM certainly are important, the application of system biological approaches have revealed more generalized mitochondrial dysfunctions, involving disturbed redox balance and oxidative stress as well as altered growth signalling pathways. Such mitochondrial driven processes may add to the pathophysiology and have recently been studied and documented in various IEMs: respiratory chain disorders (Wei et al 2009; Wu et al 2010, 2014; Elstner and Turnbull 2012; Koopman et al 2012, 2013; Zhang et al 2013), organic acidurias (Wajner and Goodman 2011; Kolker et al 2013), fatty acid oxidation disorders (Gregersen et al 2008; Gregersen and Bross 2010; Olsen et al 2013) and metabolic neuromuscular disorders (Cornelius et al 2014a). For most of these diseases, there is still no or very limited insight into the specific molecular triggers of mitochondrial dysfunction and into the cellular mechanisms that invoke chronic mitochondrial dysfunction, oxidative stress and altered signalling growth pathways. Here we summarize current knowledge of oxidative stress and mitochondrial dysfunction in IEM. We discuss how accumulation of pathological levels of reactive oxygen species (ROS) and oxidative damage may disturb healthy redox signalling between mitochondrial function and cellular repair responses, and causes disturbed cell growth control and increased stress susceptibility.

Before discussing oxidative stress in IEM, we will give a brief overview of the mechanisms by which mitochondria integrate cellular metabolism with cellular defense and repair mechanisms to maintain cellular homeostasis and health in response to physiological or environmental changes or stresses.

## Coordinated expression of the mitochondrial and nuclear genomes is critical in maintaining functional and healthy mitochondria and cell homeostasis

Reactive oxygen species (ROS) are essential for the complex relationship between the nuclear and mitochondrial genomes that was initiated a billion of years ago, when an aerobic bacterium was engulfed by an anaerobic cell and developed a symbiotic relationship. In this new Eukaryotic cell, anaerobic glycolytic energy production, through the nuclear genome and cytosolic machinery, was integrated with the oxidative mitochondrion containing its own plasmid DNA (mtDNA). Since then most of the mitochondrial genome has been transferred to the nucleus, and the mtDNA codes for only 13 subunits of the respiratory chain in addition to tRNAs and rRNAs needed for their translation. The nuclear DNA codes for the remaining respiratory chain components and other enzymes needed for oxidative metabolism like those involved in the tricarboxylic acid (TCA) cycle, fatty acid oxidation and amino acid oxidation. It also codes for proteins needed for mtDNA replication and transcription, and for proteins assisting in the import and maturation of mitochondrial proteins, and for modulating the dynamic structure of mitochondria, where healthy mitochondria form a fused threadlike structure and damaged part of mitochondria are removed by fission through a selective autophagy process called mitophagy (see below). Thus, the nuclear genome codes and controls most of proteins needed for mitochondrial structure and function, including providing the cell with energy and biosynthetic intermediates needed for cell growth and repair (reviewed in (Sanchis-Gomar et al 2014; Suliman and Piantadosi 2014)).

### ROS production and regulation of its damaging effects

**Fig. 1:**
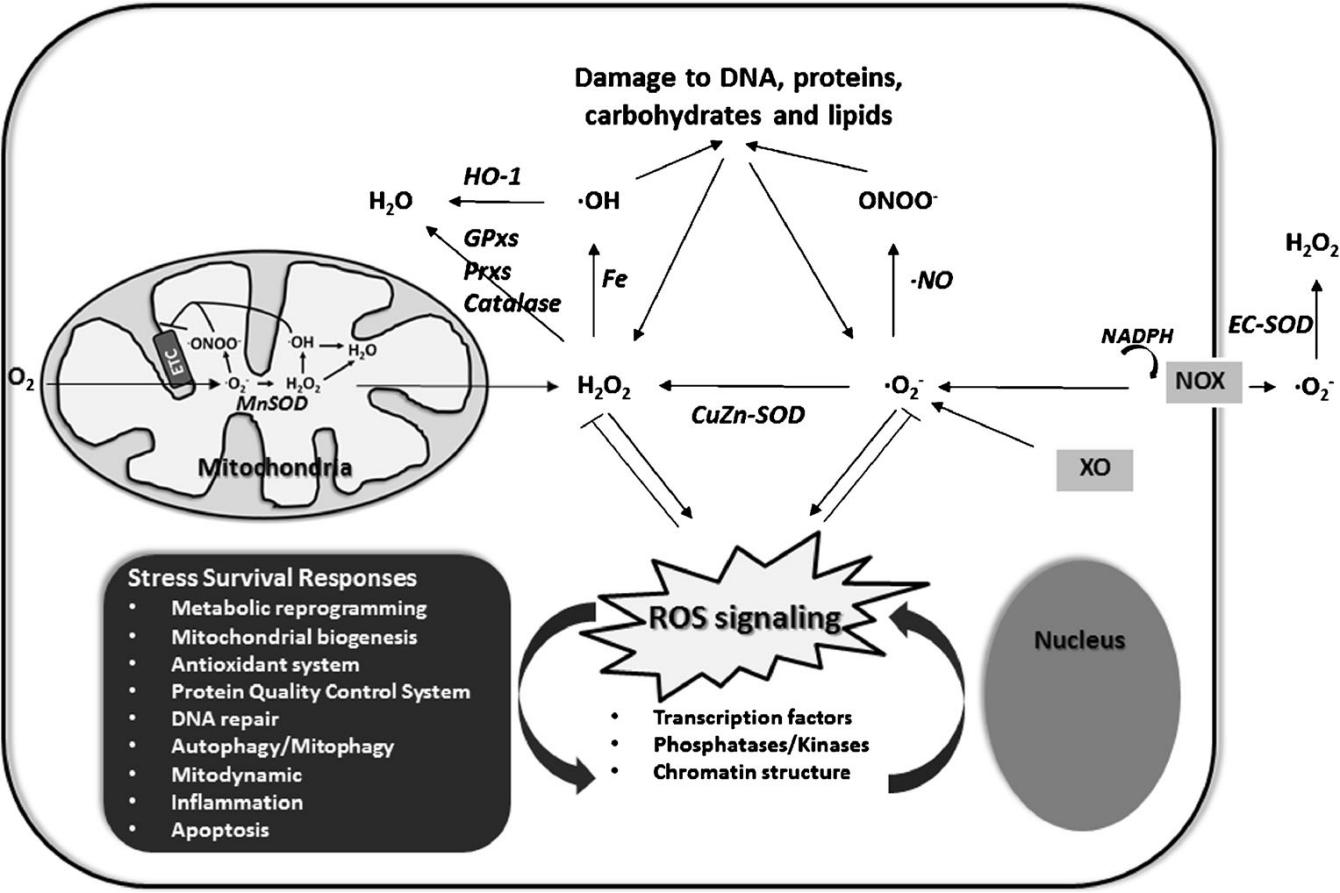
Reactive oxygen species (ROS) regulate cellular stress responses by redox regulation of transcription factors, phosphatases/kinases and chromatin structure that control nuclear gene networks to maintain cellular homeostasis and health as described in the text. Most intracellular ROS is produced inside mitochondria by the electron transport chain (ETC). The NADPH oxidases (NOX) and the xanthine oxidase (XO) also contribute to cellular ROS production. Superoxide (·O_2_^−^) is the main initial free radical species, which can be converted to other ROS and reactive nitrogen species as described in the text. Abbreviations are: GPxs; gluthathion peroxidases, Prxs; peroxiredoxines, HO-1; hemeoxygenase-1, and SOD; superoxide dismutase

Although ROS are generated from many sources, including the Nox family of NADPH oxidases, cytochrome P450 enzymes and xanthine oxidase, over 90 % of intracellular ROS is produced inside mitochondria. ROS, in the form of superoxide radical anions (O_2_^.-^) are produced by incomplete reduction of oxygen or by leak of electrons from the respiratory chain, mostly complex I and III. However, also other mitochondrial enzymes like complex II, the electron transfer flavoprotein (ETF) and its ubiquinone oxidoreductase (ETF:QO), α-ketogluterate dehydrogenase and glycerol-3-phosphate dehydrogenase have been implicated in ROS production (Dröse and Brandt 2012; Goncalves et al 2015). Whereas all these mitochondrial enzymes release superoxide in the matrix, complex III releases superoxide both in the matrix and the intermembrane space. Superoxide is highly reactive and in toxic levels can oxidize and damage the structure and function of other molecules in the cell or react with nitric oxide (·NO) that leads to the formation of the highly deleterious peroxynitrite species (ONOO^−^). To prevent these harmful reactions, superoxide is rapidly converted into hydrogen peroxide (H_2_O_2_) by the superoxide dismutases (SOD). In the presence of transition metals such as iron or cupper, hydrogen peroxide can be further reduced to the highly reactive hydroxyl radical (^.^OH) by the Fenton reaction, or be detoxified to water by peroxiredoxins (Prxs), glutathione peroxidases (GPxs) or catalase. All these ROS scavenger enzymes function to limit ROS or reactive nitrogen species (RNS) induced oxidative damage, but important for the content of this review, also to buffer cellular ROS species (especially H_2_O_2_), and bring them to a level at which they function as signalling molecules between mitochondrial function and redox activation of cell stress protective pathways (Bolisetty and Jaimes 2013) (Fig. 1).

H_2_O_2_ can diffuse through membranes to the cytosol and oxidize thiol residues in stress regulatory proteins — such as transcription factors, kinases and phosphatases — to control their activity in a concentration dependent manner (Marinho et al 2014). Likewise, at increased ROS levels, electrophiles — produced by oxidation of unsaturated fatty acids resulting in lipid peroxidation products — can transfer their oxidation state to proteins and regulate their function through the oxidation of cysteine residues on the recipient proteins (Higdon et al 2012; Kansanen et al 2012). In that way, ROS and electrophiles are parts of a redox signalling system involved in the regulation of mitochondrial quality control pathways and overall cell repair (Fischer et al 2012; Kotiadis et al 2014; Levonen et al 2014). The activated pathways control the activity of the antioxidant system and metabolic reprogramming to decrease ROS production and also control the activity of DNA repair, protein quality control systems and autophagy/mitophagy to hinder damaged DNA, proteins and mitochondria to cause further ROS production and cell death. If the ROS increases above a required level, where these redox-sensitive stress response pathways can no longer cope with the increased ROS, apoptotic cell death pathways will be activated to hinder necrosis and organ failure as will be discussed further below.

### Integrated gene networks link mitochondrial biogenesis and function to cell defense and repair systems

**Fig. 2:**
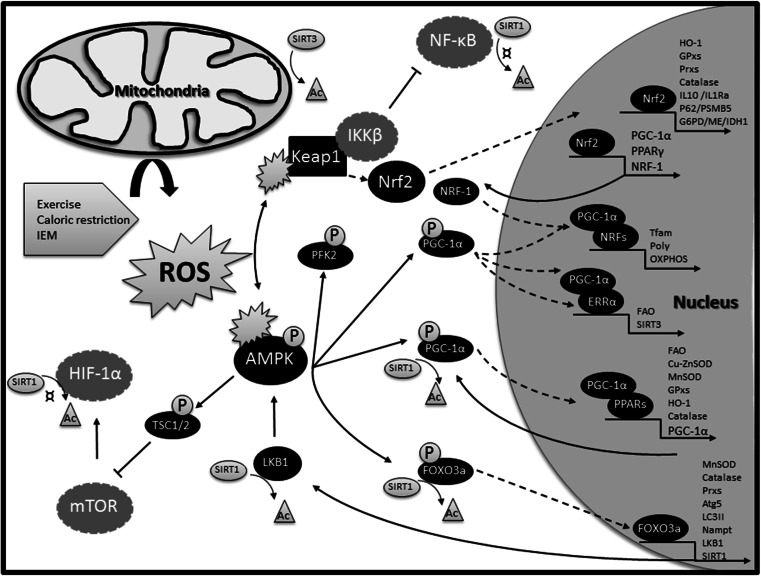
An integrated gene network links mitochondrial biogenesis to cell repair functions. AMPK and Nrf2 are central players in activating this gene network during oxidative stress, as both proteins are activated by redox modification of critical cysteine residues as described in the text. Nrf2 directly binds to promoters of a number of antioxidant (HO-1, GPxs, Prxs, catalase, SOD), anti-inflammatory proteins (IL10, IL1Ra), as well as autophagy (p62) and proteasomal (PSMB5) proteins, and also to proteins (G6PD, IDH1, ME), needed for synthesis and regeneration of the antioxidant NADPH as described in the text. Sirtuin-1 (SIRT1) activates AMPK via de-acetylation of LKB1, which subsequently triggers AMPK activation by phosphorylation. Activated AMPK phosphorylates downstream targets like PGC-1α, which upon de-acetylation binds to and co-activates transcription factors involved in mitochondrial biogenesis and dynamics such as the nuclear respiratory factors (NRF-1 and NRF-2). NRF-1 in turn binds to and modulates expression of other factors such as mitochondrial DNA polymerase (Polγ) and mitochondrial transcription factor A (Tfam), which regulate mtDNA replication and transcription, and also binds to and activates a number of genes required for oxidative phosphorylation (OXPHOS) through expression of respiratory chain components. PGC-1α can also activate the nuclear estrogen-related receptors (ERRα), which binds to the promoters of fatty acid oxidation enzymes (FAO) and to the promoter of the NAD^+^-dependent deacetylase, sirtuin-3 (SIRT3), which is required for post-translational activation of a number of metabolic and antioxidant enzymes located inside mitochondria. PGC-1α can also directly activate the expression of antioxidant and FAO enzymes by co-activating PPARs. PPARs comprise three members; PPARα, PPARβ and PPARγ, each responsible for tissue specific activation of FAO and antioxidant proteins, albeit with some overlap. When activated any of the PPARs can induce the expression of PGC-1α. Moreover, AMPK can phosphorylate the FOXO3a upon oxidative stress to promote its nuclear translocation and expression of antioxidants (MnSOD, catalase, Prxs) and autophagy (Atg5, LC3II) proteins. FOXO3a also binds to the promoter of the AMPK-activating protein kinase LKB1, the SIRT1 promoter and to the Nampt gene promoter and induces NAD^+^ synthesis and further AMPK activation. AMPK also up-regulates glycolysis by increasing fructose-2,6-biphosphate concentrations through phosphorylation of PFK2. Finally, AMPK can phosphorylate tuberous sclerosis complex 2 (TSC2) and thereby inhibit mTOR, which is a negative regulator of authophagy and an activator of HIF-1α. Thus, to sustain energy requiring repair function during cellular stress, AMPK activates mitochondrial biogenesis and inhibits energy-demanding cellular functions, such as cell growth, and immune responses. The inhibitory effects of AMPK on immune responses are likely to be indirect and governed by downstream mediators such as SIRT1 mediated de-acetylation and inactivation of NF-κB. NF-κB signalling is also kept in an inactive state by Nrf2. Nuclear translocation of NF-κB requires activation by an IKKβ kinase, which like Nrf2 is targeted for proteasomal degradation by Keap1. When Nrf2 is released from Keap1 by moderate increases in oxidative stress, there is an increase in unbound Keap1 available for IKKβ binding, thus inhibiting the expression of NF-κB target genes. Abbreviations not explained in the text are: Atg5; autophagy protein 5, G6PD; glucose-6-phosphate dehydrogenase, IDH1; isocitrate dehydrogenase 1, LC3II; the phosphatidylethanolamine form of microtubule-associated protein 1A/1B-light chain 3, ME; malic enzyme, PFK2; phosphofructokinase 2, and PSMB5; proteasome subunit beta type 5. The symbol “¤” indicates that the proteins are inactivated by SIRT1 mediated de-acetylation. Activated proteins are in *black* and repressed once in *grey*

The distinct actors of the repair systems — such as antioxidant enzymes, DNA repair enzymes, chaperones and proteases — require energy and other mitochondrial products like NAD^+^ for their functional activity. Therefore, the cell needs to adapt or expand its mitochondrial population during episodes of cell damage by the induction of mitochondrial biogenesis (Piantadosi and Suliman 2012). Mitochondria exist as a tubular network in the cells, and they are not generated de novo. Rather, healthy mitochondria in the network are stimulated to proliferate, while defective mitochondria are selected and removed by mitophagy (Youle and van der Bliek 2012; Archer 2013). The peroxisome proliferator-activated receptor γ (PPARγ) coactivator 1α protein (PGC-1α) is a major upstream regulator of the gene network that controls mitochondrial biogenesis. PGC-1α is activated and regulated by a multitude of mechanisms (Scarpulla et al 2012; Suliman and Piantadosi 2014). For the present discussion, we will focus on its potential activation during oxidative conditions (Fig. 2).

The serine/threonine AMP-activated protein kinase (AMPK) inhibits ATP consuming pathways and activates ATP generating pathways, like mitochondrial biogenesis, fatty acid oxidation and glycolysis (Shirwany and Zou 2014). AMPK is an upstream activator of PGC-1α. It has been shown that AMPK upon oxidation of certain cysteines by H_2_O_2_ changes its conformation, and then may be autophosphorylated and activated in an AMP-dependent or AMP-independent manner (Wu and Wei 2012; Hagenbuchner and Ausserlechner 2013). The phosphorylated AMPK then phosphorylates downstream transcription factors like PGC-1α and the Forkhead box O (FOXO3a). FOXO3 belongs to the O subclass of the forkhead family of transcription factors, which are involved in protection from oxidative stress. The phosphorylated proteins then translocate to the nucleus and FOXO3a binds directly to the promoters of antioxidant and autophagy genes (Kops et al 2002; Chiribau et al 2008; Li et al 2009; Wu et al 2014). PGC-1α co-activates transcription factors like the nuclear respiratory factors (NRFs), which are involved in mitochondrial biogenesis and dynamics and estrogen-related receptor α (ERRα), which activates fatty acid oxidation enzymes. PGC-1α also co-activates peroxisome proliferator-activated receptors like PPARα, PPARβ and PPARγ, which bind to promoters of fatty acid oxidation and antioxidant genes (Polvani et al 2012; Scarpulla et al 2012; Kemper et al 2014) as illustrated in Fig. 2. FOXO3a can also bind to the nicotinamide phosphoribosyltransferase (Nampt) gene promoter and induce its transcription. Nampt induction results in increased NAD^+^ levels, which in turn support the de-acetylation activity of sirtuins. Sirtuins are essential parts within nuclear-mitochondrial communication, where SIRT1 — located in the nucleus — de-acetylates and activates PGC-1α and FOX03a, and also indirectly activates AMPK by de-acetylation and activation of the AMPK-activating protein kinase LKB1. The mitochondrial located SIRT3 in turn de-acetylates and activates many of the mitochondrial biogenesis and repair enzymes expressed by these transcription factors, such as respiratory chain and fatty acid oxidation enzymes, MnSOD and isocitrate dehydrogenase 1 (reviewed in (Houtkooper et al 2012; Brenmoehl and Hoeflich 2013)).

Another important transcription factor, which becomes activated upon oxidative stress is Nrf2. Under normal conditions, Nrf2 is kept in the cytoplasm by Kelch like-ECH-associated protein 1 (Keap1) and Cullin 3 (Cul3), which lead to ubiquitination and degradation of Nrf2 by the proteasome. Oxidative stress or electrophilic stress modify certain cysteine residues in Keap1, and thereby inhibit Keap1-Cul3 mediated degradation of Nrf2, which then translocates to the nucleus and activates a number of antioxidative and anti-inflammatory genes. Nrf2 can also bind and activate NRF-1 expression and perhaps PGC-1α and PPARγ upon electrophilic or oxidative stress, thereby connecting antioxidant functions with mitochondrial biogenesis (Piantadosi and Suliman 2012; Polvani et al 2012; Baldelli et al 2013).

The gene networks that connect the processes of protein quality control and mitophagy to mitochondrial biogenesis are less well characterized. However, Nrf2 activation has been reported to regulate the removal of misfolded proteins as well as damaged mitochondria by increasing the expression of proteasome subunits (PSMs) of the 26S proteasome (Kwak et al 2003), and expression of the autophagy adaptor protein p62 (Jain et al 2010), which is needed for the selective removal of damaged mitochondria by mitophagy (Vives-Bauza et al 2010). At the same time, p62 has been shown to interact with the Keap1-binding site of Nrf2, resulting in activation of Nrf2 and its downstream targets (Komatsu et al 2010). Moreover, the nuclear respiratory chain factor NRF-2 has been suggested to bind to the proximal promoter of the mitochondrial protease LON (Pinti et al 2011).

As illustrated in Fig. 2, the activity of AMPK and the expression of PGC-1α, FOXO3a, PPARs, Nrf2 and sirtuins seems to be linked by a positive feedback loop that ensures coordinated expression of antioxidant and other survival genes, as long as oxidative stress persists. At the same time, the genes exert an anti-inflammatory action, inhibiting the pro-inflammatory transcription factor, nuclear factor Kappa-light-chain-enhancer of activated B cells (NF-κB) (Salminen et al 2011; Polvani et al 2012). The activity of NF-κB is also inhibited by AMPK-mediated phosphorylation and activation of TSC1/2, which keeps mammalian target of rapamycin (mTOR) in an inactive state as discussed in more details below.

Thus, to support energy demanding processes of cell repair, a fine-tuned coordination of mitochondrial biogenesis to the cell’s antioxidant, anti-inflammatory, protein quality control and autophagy functions has been developed. This integrated network also reflects that ROS is a natural byproduct of mitochondrial respiratory chain activity, and consequently that the development of mechanisms to handle ROS from cellular oxygen metabolism has been essential through the evolution of the eukaryotic cell.

**Fig. 3:**
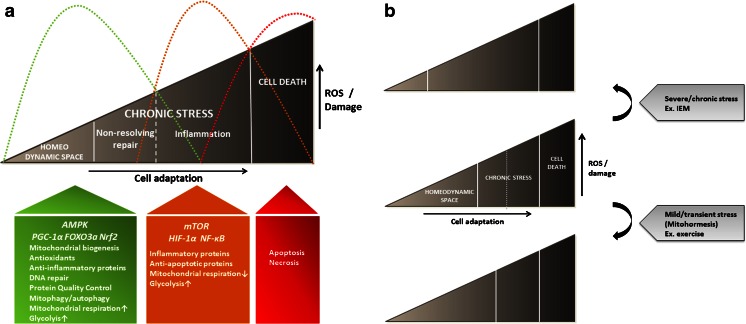
The ROS triangle model links increasing ROS and damage to chronic stress adaptation with non-resolving repair responses (*green graphic*), or compromised repair responses that drive a more pro-inflammatory environment (*orange graphic*). The antagonistic cell stress responses are linked to distinct cellular metabolism through redox-sensitive nutrient-sensing signalling growth pathways that control a Warburg-like shift from mitochondrial respiration (AMPK/PGC-1α) towards mostly cytosolic glycolysis (mTOR/HIF-1α). ROS, but also NAD^+^, are the most important mitochondrial signalling molecules that drive the transition from one stage of the triangle to another as discussed in the text and illustrated in Fig. 4. When oxidative stress becomes too high to allow cell stress adaptive redox signalling, apoptosis and cell death are induced (*red graphic*) (**a**). In the sick cell, chronic non-resolving repair responses or inflammatory responses will dominate depending on the duration and/or level of ROS load. In the healthy cell, well-controlled physiological levels of ROS allow healthy redox signalling and dynamic cell stress responses to ensure that inflammatory and damaging cell responses are followed by repair responses to restore homeostasis. The ROS range, at which dynamic healthy redox signalling is taking place, to regulate transient physiological changes or stressors like cell growth/differentiation and inflammation/repair, is called the homeodynamic space. Mild and transient oxidative stress, such as exercise and caloric restriction, increases the homeodynamic space by boosting repair responses, and prevent chronic disease development. IEM and other persistent ROS-inducers decrease the homeodynamic space, making the cells more prone to chronic disease development (**b**)

Mild and transient oxidative stress conditions or energy demanding processes, such as exercise and caloric restriction, can induce mitochondrial biogenesis with its integrated repair gene networks, and thereby produce a larger and healthier population of mitochondria (Lopez-Lluch et al 2006; Safdar et al 2011; Milisav et al 2012; Piantadosi and Suliman 2012; Aquilano et al 2013; Rodell et al 2013). Such cells are more adapted to handle a subsequent event of energy crisis or damaging ROS levels, as for example in connection with an infection or ischemia. This concept that low levels of a mitochondrial damaging agent improve systemic defense mechanisms by inducing an adaptive response has been named mitochondrial hormesis or mitohormesis (Demirovic and Rattan 2013; Ristow and Schmeisser 2014). Thus, in healthy cells mitochondrial biogenesis, in combination with a basal level of mitophagy, is a beneficial and essential process that maintains or improves healthy cell stress responses (Baldelli et al 2014). However, when mitochondria accumulate oxidative damage — for example under pathological conditions in cells with inborn errors of metabolism — the fine-tuned redox regulation of integrated mitochondrial biogenesis and repair systems may be disturbed, and contribute to the accumulation of oxidative stress and cell damage as illustrated in Fig. 3a and b and further discussed below.

## Oxidative stress causes maladaptive mitochondrial repair responses and chronic cell stress in inborn errors of metabolism

ROS and cellular signs of oxidative stress in IEM have been documented in a number of studies of patient cells, model cells and animals. A detailed review of these data is outside the scope of this review, and the reader is referred to comprehensive review papers that discuss this topic (Gregersen et al 2008; Wei et al 2009; Gregersen and Bross 2010; Wu et al 2010, 2014; Wajner and Goodman 2011; Koopman et al 2012, 2013; Kolker et al 2013; Olsen et al 2013; Cornelius et al 2014a).

The exact mechanisms by which IEM produce ROS are not known. Defects in mitochondrial DNA or nuclear DNA, that directly affect respiratory chain function or its supercomplex integrity, may cause ROS by electron leak or by incomplete reduction of oxygen, as described for gene defects in complex I (Patsi et al 2008; Sharma et al 2011), complex II (Guo and Lemire 2003; Huang and Lemire 2009) and the electron transfer flavoproteins (Cornelius et al 2012, 2013; Rodrigues and Gomes 2012). Other gene defects, not directly affecting respiratory chain enzymes, may cause accumulation of misfolded variant proteins or toxic metabolites that can interact with and disturb the structural organization and function of the respiratory chain enzymes or their supercomplexes, and in that way initiate the production of ROS as discussed in (Gregersen and Bross 2010; Wajner and Goodman 2011; Olsen et al 2013). Because of its proximity to the site of ROS production, and its lack of protecting repair enzymes and histone molecules, mtDNA is particularly vulnerable to oxidative damage, and when affected can cause further respiratory chain dysfunction (Wu et al 2010). It is likely, that mtDNA damage, together with moveable lipid peroxidation products, can propagate and amplify a vicious cycle of oxidative damage and ROS production in the mitochondrial membrane network with increasing damage to its protein components.

These mechanisms can cause functional decline in respiratory chain function, sometimes with a metabolic reprogramming towards a more glycolytic energy production, which seems to be a hallmark of many IEM and in particularly respiratory chain disorders (Smeitink et al 2001; Schapira 2006; Wajner and Goodman 2011; Kolker et al 2013; Koopman et al 2013). As a consequence of this metabolic shift, which is known as the Warburg effect, pyruvate from glycolysis is mostly converted to lactate instead of being shuttled into the mitochondrion for further conversion to acetyl-CoA and oxidation through the tricarboxylic acid (TCA) cycle and oxidative phosphorylation. Although it is well established that respiratory chain dysfunction and energy crisis can induce metabolic reprogramming, an increasing amount of data support that Warburg-like shifting is essential for cellular adaptation to oxidative stress (Grant 2008; Wu and Wei 2012; Holley et al 2013).

Below we will discuss recent findings in IEM, supporting the hypothesis, that the Warburg effect flow in the shade of oxidative cell stress responses through integration with nutrient-sensing signalling growth pathways. The working model in Fig. 3a is a modified version of the ROS triangle model that we presented in a recent review, based on mitochondrial proteomic and cell stress studies on primary skin fibroblasts derived from patients with various inborn errors of fatty acid oxidation (Olsen et al 2013). According to the working model in Fig. 3a, the first line of adaptive stress responses is fueled by an AMPK-induced metabolic reprogramming in which mitochondrial biogenesis, in combination with increased glycolysis, support bioenergetic supporting molecules for the repair systems. The second line of stress responses is characterized by a marked decrease in mitochondrial respiration — most likely induced by a pseudo-hypoxic HIF-1α response — to inhibit further electron leakage and ROS production from a damaged respiratory chain, which would otherwise induce apoptosis or necrosis. The consequences of the second line of stress responses are compromised cell repair responses that drive a more pro-inflammatory environment. ROS is the most important triggering factor, and it also drives the transition from one stage of the triangle to another. We will argue for the model using examples primarily from our own and other’s studies of multiple acyl-CoA dehydrogenation deficiency (MADD) caused by mutations in the electron transfer flavoprotein:ubiquinone oxidoreductase (ETF:QO). We will also include elegant and illustrative studies carried out by Yau-Huei Wei’s lab on fibroblasts derived from patients with myoclonic epilepsy with ragged-red fibres (MERRF) due to the common mtDNA 8344A>G tRNA^Lys^ mutation.

### Coordination of metabolic and repair functions to cope with energy requirement and oxidative stress; the first line of adaptive stress responses

Multiple acyl-CoA dehydrogenation deficiency (MADD) is a disorder of mitochondrial metabolism in which mutant genotypes have been linked to different ROS levels and clinical phenotypes with secondary respiratory chain deficiency. Primary skin fibroblasts from MADD patients may therefore serve as good models to study how different levels of endogenous ROS regulate mitochondrial cell stress pathways and cause respiratory chain dysfunction and disease.

MADD is caused by gene variations in electron transfer flavoprotein (ETF) or its ubiquinone oxidoreductase (ETF-QO). These two enzymes link a number of fatty acid and amino acid dehydrogenation reactions to ATP production in the respiratory chain, by delivering electrons to coenzyme Q10 (CoQ10) and complex III (Watmough and Frerman 2010). A relationship between genotype and phenotype has been documented in which patients with multi-organ failure and neonatal or early childhood death carry gene defects that cause severely decreased amount of protein and almost total lack of enzyme activity. These patients are named “severe MADD” or S:MADD. Patients with later-onset of disease and predominantly muscle symptoms carry gene defects that give rise to milder folding defective proteins with enzyme activities that can be modulated, and sometimes rescued by FAD co-factor treatment in the form of riboflavin (Goodman et al 2002; Olsen et al 2003, 2007; Cornelius et al 2012). These patients are named “riboflavin-responsive MADD” or RR:MADD. Muscle biopsies from later-onset patients often show secondary respiratory chain and CoQ10 deficiencies combined with lipid storage (Gempel et al 2007; Olsen et al 2007; Wen et al 2010; Grunert 2014).

The ETF/ETF-QO complex — with its integrated flavin and iron-sulfur clusters — has the capacity to produce ROS during fatty acid oxidation (Goncalves et al 2015; St-Pierre et al 2002), and at increased levels when fatty acid oxidation is disturbed by mutations in ETF/ETF-QO (Rodrigues and Gomes 2012; Cornelius et al 2013). By over-expression studies in HEK-293 cells, we recently showed that mild folding defective variant ETF-QO proteins produce more ROS than severe folding defective and degraded ETF-QO proteins (Cornelius et al 2013). In accordance with these findings, a proteomic study of fibroblasts from a S:MADD patient and total lack of ETF-QO protein revealed signs of mild oxidative stress by activation of cell repair pathways with increased expression of antioxidant enzymes like MnSOD, peroxiredoxin and glutathione reductase, and increased levels of a number of heat shock proteins (Hsp90, Hsp70, Hsp78 and Hsp60), which are part of the cellular protein quality control system. This up-regulation of cellular repair systems was accompanied by increases in glycolytic enzymes and in cytosolic isocitrate dehydrogenase 1, whereas fatty acid oxidation enzymes were down-regulated and respiratory chain enzymes showed a more mixed regulation (Rocha et al 2011). These studies confirm well earlier studies in a S:MADD Zebrafish model, which lacks ETF-QO protein due to an endogenous defect. In this Zebrafish model, increased superoxide production is associated with transcriptional up-regulating of glycolytic enzymes along with the antioxidant enzymes glutathione reductase and uncoupling protein 3, and the heat shock proteins Hsp90 (Song et al 2009).

As mentioned, this up-regulation of glycolysis (Warburg-like effect) is a general finding in IEM and is essential for their adaptation, not only to energy crisis, but also to oxidative stress. Oxidative stressed cells take up more glucose to fuel them into the pentose phosphate pathway for production of NADPH by glucose-6-phosphate dehydrogenase. Activation of NADPH producing pathways, which besides glucose-6-phosphate dehydrogenase also include cytosolic isocitrate dehydrogenase 1 and malic enzyme, is a hallmark of the Warburg phenotype (Vander Heiden et al 2009). NADPH is an essential co-factor for glutathione and thioredoxin recycling, and thereby for H_2_O_2_ detoxification by glutathione peroxidase and peroxiredoxin, which are regulated by the Nrf2 system. Actually, many enzymes needed for synthesis and regeneration of NADPH are Nrf2 targets themselves, again illustrating a cellular need for coordination of antioxidant defense mechanisms with metabolism (Heiss et al 2013) (Fig. 2). Accordingly, Yau-Huei Wei’s lab has shown that cells from MERRF patients, with the common mtDNA 8344A>G tRNA^Lys^ mutation, show increased ROS, and decreased NADPH and cell survival when glycolysis is inhibited by genetic knockdown of AMPK or glucose depletion, indicating that up-regulation of glycolysis is an oxidative stress protective mechanism (Wu and Wei 2012). Similarly, fibroblasts, derived from patients with genetic defects of fatty acid oxidation enzymes, and challenged with the ROS-inducing drug menadione, die faster when depleted of glucose (Zolkipli et al 2011). Importantly, Yau-Huei Wei’s lab showed that AMPK can be activated in a H_2_O_2_-dependent and AMP-independent manner (Wu and Wei 2012), and that activated and phosphorylated AMPK levels in MERRF fibroblast cells correlate with increased phosphorylation and expression of a number of downstream AMPK targets, such as PGC-1α, and FOXO3a, which are transcription factors that control mitochondrial biogenesis, glycolysis and cell repair pathways, respectively as described above (Wu and Wei 2012; Wu et al 2014). Accordingly, in addition to activation of glycolytic and antioxidant enzymes, MERRF cells do also show accumulation of autophagosomes and increased mitophagy, which can be restored by ROS scavenger treatment in the form of CoQ10 (De la Mata et al 2012). The level of the phosphorylated and active forms of AMPK is also significantly increased in the MADD zebrafish (Song et al 2009). Together with the observed increases in glycolytic and antioxidant genes, this indicates that MADD cells may activate a similar nuclear gene network as observed in MERRF cells.

All together these studies suggest that a first line of adaptive stress responses to mild oxidative stress could be ROS-mediated activation of AMPK, which links metabolic reprogramming and mitochondrial biogenesis with activation of stress repair pathways to protect the cell from oxidative damage. Because of the inborn gene defects, the repair responses may be non-resolving and persistent resulting in over-proliferating mitochondria and the so-called ragged-red fibers phenotype, which is a pathological hall mark of many mtDNA diseases, including MERRF (Wu et al 2014). Chronic over-proliferating mitochondria have been linked to cardiomyopathies, which are common in IEM (Sebastiani et al 2007; Bates et al 2012). Another consequence of non-resolving mitochondrial biogenesis, during oxidative stress, could be increased risk of molecular damage spreading into the mitochondrial network, because of the ongoing mitochondrial content mixing during repeated and prolonged fission-fusion events. This may be one explanation for the clonal expansion of mutant mitochondrial DNA in respiratory chain disorders (Figge et al 2013) and other diseases/conditions associated with mitochondrial dysfunction such as heart failure and aging (Wei et al 2009; Dai et al 2010; Ahuja et al 2013). As time progresses, this could also contribute to increasing oxidative stress and increasing risk of irreversible structural damage that prevent redox activation of cell repair pathways as discussed below, and force the cell into the second line of adaptive stress responses.

### Decreased mitochondrial respiration and repair functions to cope with redox stress and prevent cell death; the second line of adaptive stress responses

With age and senescence, and under chronic disease conditions, cells have compromised repair mechanisms, or seem to lose their ability to induce them during acute stress. For example MnSOD has been found decreased in a number of studies of patient cells, model cells and animals with chronic diseases, like diabetes, cardiovascular diseases, cancer and kidney disease (Scott et al 2010; Bauer et al 2011; Charniot et al 2011; Mohelnikova-Duchonova et al 2011; Olsson et al 2011). Similarly the Lon protease (Ngo et al 2013) and autophagy and mitophagy processes (Salminen and Kaarniranta 2009; Bolisetty and Jaimes 2013), which are essential for elimination of damaged mitochondrial proteins or organelles, are decreased in the same or other diseases with chronic cell stress, and so are anti-inflammatory proteins (Liu et al 2012; Tabas and Glass 2013). Interestingly, mitochondrial biogenesis (Sanchis-Gomar et al 2014) and sirtuins (Yechoor et al 2004; Palacios et al 2009; Sundaresan et al 2009) are also decreased, which as mentioned above, plays important roles in coordinating the transcriptional and post-translational regulation of mitochondrial metabolism and antioxidant function in response to energy and oxidative stress.

The mechanisms that drive these blunted repair responses are still unknown and a subject of intensive research. Some lessons can be learned from RR-MADD and other IEM. Most of the patients suffering from RR-MADD have secondary respiratory chain and CoQ10 deficiency (Gempel et al 2007; Olsen et al 2007; Wen et al 2010; Cornelius et al 2013; Grunert 2014). Many of the gene defects are located near the site of ubiquinone reduction, and our data suggest that the ETF-QO ubiquinone-binding site can become a source of relative intensive superoxide production when the ETF-QO protein misfolds due to gene defects (Cornelius et al 2012, 2013).

In contrast to what has been observed in S:MADD cells, RR:MADD cells seem to have decreased levels of key proteins involved in mitochondrial repair mechanisms. In a study, in which fibroblast cells, derived from six unrelated RR-MADD patients, were cultured and compared to that of control fibroblasts, RR-MADD cells showed decreases in the protein levels of the antioxidant enzyme MnSOD, the protein quality control enzyme Lon, and the p62 protein — which is needed for recognition and selection of oxidative damage mitochondria for autophagy degradation. Also mitofusin 2, which is one of the key regulators of mitochondrial fusion events during mitochondrial biogenesis and dynamics, was decreased. Accordingly, the mitochondrial network had a fragmented morphology (Cornelius et al 2014b). In addition to decreased fatty acid oxidation enzymes, these RR:MADD cells also showed decreased levels of SIRT3.

SIRT3 controls the activity of mitochondrial oxidative phosphorylation and antioxidant function by direct de-acetylation of key metabolic and antioxidant enzymes as described above, and also indirectly by decreasing ROS-induced stability of hypoxia-inducible factor-1α (HIF-1α). HIF-1α regulates the expression of a gene network leading to the Warburg effect and down-regulation of respiratory chain function in cancer cells (Bell et al 2011; Finley et al 2011; Schumacker 2011; Huang et al 2014).

Interestingly, treating the RR-MADD cells with CoQ10 decreased the amount of ROS production (Cornelius et al 2013), and to some extent reverted the decreased levels of Lon, p62 and mitofusion 2, but did not significantly affect the levels of Sirt3 and MnSOD (Cornelius et al 2014b). As described above and illustrated in Fig. 2, MnSOD and SIRT3 — along with expression of fatty acid oxidation enzymes — are regulated by the master regulator of mitochondrial biogenesis PGC-1α through its binding to and co-activation of the PPARs and ERRα transcription factors, respectively. MnSOD is also controlled by the FOXO3a transcription factor — which, like PGC-1α, becomes phosphorylated and activated upon ROS-induced activation of AMPK in MERRF cells (Wu et al 2014). The transcriptional regulation of Lon and p62 is not well known. However, it is likely that their expression is also associated with mitochondrial biogenesis and oxidative stress as discussed above and illustrated in Fig. 2.

Thus, it is likely that in RR:MADD cells, impaired activation of AMPK/PGC-1α/FOXO3a (the first line of adaptive stress response) results in blunted repair responses, as shown in cells from patients with Friedreich’s ataxia. These patients suffer from mitochondrial dysfunction and oxidative stress due to mutant frataxin, which is involved in the regulation and maturation of iron-sulfur protein activities (Cornelius et al 2014a) and references herein). It has been shown in patient skin fibroblasts and in a frataxin knock-out mouse that the decreased amount of PGC-1α results in decreased MnSOD and a loss of MnSOD activation in response to oxidative stress. This response can be restored by AMPK and PPARγ agonists (Marmolino et al 2010).

Thus, severe or persistent oxidative stress load may induce a state of chronic oxidative stress with compromised oxidative stress protective responses that obviously will make the cells more susceptible for further stress-induced disease and cell death. This innate predisposition to stress induced cell death is illustrated in a study in which cultured fibroblasts from patients with genetic deficiencies of various fatty acid oxidation enzymes, as compared to control cells, suffered from early death when challenged with the ROS-inducing anticancer drug menadione (Zolkipli et al 2011). Interestingly, in the same study survival was increased when the cells were treated with antioxidants or the PPAR agonist bezafibrate, which boosts PGC-1α mediated mitochondrial respiration and antioxidant function. This suggests that also in these cells mitochondrial biogenesis and stress tolerance might be limited by ROS induced AMPK/PGC-1α/FOXO3a/signalling defects. Like bezafibrate, resveratrol is known to increase mitochondrial biogenesis and antioxidant function; although in a more indirect manner by binding to and stimulating complex I, thereby increasing the NAD^+^ and sirtuin-mediated activation of mitochondrial respiration and antioxidant function (Desquiret-Dumas et al 2013). Accordingly, not only bezafibrate (Djouadi et al 2005; Gobin-Limballe et al 2007; Bonnefont et al 2009, 2010; Li et al 2010; Casarin et al 2012; Yamaguchi et al 2012), but also resveratrol (Lopes et al 2014; Aires et al 2014; De Paepe et al 2014,), and the AMP analogue and AMPK activator 5-aminoimidazole-4-carboxamide ribonucleoside (AICAR) (Golubitzky et al 2011) have been shown to boost fatty acid oxidation and respiratory chain capacity in cells from patients with inborn errors of metabolism. Although oxidative stress and mitochondrial stress responses were not investigated, these data suggest that mitochondrial biogenesis — and thereby activation of cellular repair responses through the AMPK/PGC-1α/FOXO3a/Nrf2 axis — is disturbed by chronic oxidative stress, and respiratory chain dysfunction, and that increased mitochondrial biogenesis can be beneficial for these IEM.

AMPK constitutes together with mTOR the major signalling nodes guiding intrinsic and extrinsic stress responses, respectively. While AMPK favours glycolysis, coupled to mitochondrial respiration, to generate energy for stress repair mechanisms, mTOR switches on ATP-consuming anabolic pathways like the synthesis of proteins, lipids and cholesterol by activating among others HIF-1α to drive cellular growth and proliferation in response to extern growth stimuli, like nutrients and infecting pathogens (Laplante and Sabatini 2012). So, these two signalling pathways respond to stress signals by antagonistic transcriptional and metabolic pathways and activation of one system prevents the activity of the other. Key to this reciprocal regulation is AMPK-mediated phosphorylation and activation of Raptor and the tuberous sclerosis complex 2 (TSC2), which keep mTOR in an inactive state. Likewise, whereas sirtuin-mediated de-acetylation is needed for the activation of AMPK (through de-acetylation of LKB1) and its down-stream targets, SIRT1-mediated de-acetylation inactivates HIF-1α activity (Lim et al 2010) and NF-κB (Kauppinen a salminen a al 2013) (Fig. 2). Accordingly, primary cell cultures with impaired AMPK signalling due to diverse IEM up-regulate the mTOR/HIF-1α pathway (Zhang et al 2013).

It will be interesting to investigate if pro-inflammatory responses are activated in IEM tissue with compromised repair responses in as much as decreased activations of these (ex. Nrf2- and AMPK-connected pathways) theoretically should force a more pro-inflammatory and pro-oxidant environment. Such an environment can in itself activate and be sustained by NF-κB-linked pro-inflammatory responses, which are transactivated by increased HIF-1α activity and decreased AMPK (Salminen et al 2011; Liu et al 2012; Piantadosi and Suliman 2012). Dysfunctional mitochondria may also release damage-associated molecular patterns (DAMPs), which can stimulate inflammasomes and a systemic inflammatory response in which NF-κB activation plays a priming role (Zhang et al 2010; Zhou et al 2011). In fact it has recently been shown, in Friedreich’s ataxia patient cells and in a frataxin knock-out mouse, that such cells have increased levels of various pro-inflammatory prostaglandins and are more sensitive to inflammatory stimulus (Hayashi et al 2014). Pro-inflammatory responses have been associated with a number of other chronic diseases such as diabetes, obesity, atherosclerosis and neurodegenerative diseases (Tabas and Glass 2013). The stress responses of acute inflammation — induced by infection/sepsis or tissue injury — are similar to those of chronic inflammation with activation of the mTOR/HIF-1α/NF-κB axis. However, the non-resolving and simmering nature of chronic inflammation differs from the intense acute responses, which switch to repair responses within days/weeks to restore homeostasis. The mechanisms responsible for this are unknown. It has been suggested that proximal sensing and signalling mechanisms for inducing chronic versus acute inflammation may differ (Liu et al 2012). Mitochondrial ROS could be the triggering factor of chronic stress and inflammation as discussed below, and as such, treatment of chronic inflammation should be directed towards preventing accumulation of ROS and its associated damage.

### Triggers of chronic cell stress and transition from one stage to the next

Lack of, or decreased amount of mitochondrial signalling molecules, especially NAD^+^ — which through sirtuin mediated de-acetylation activates the AMPK/PGC-1α/FOXO3a signalling pathways — has been put forward to explain the compromised mitochondrial function and repair mechanisms in chronic disease conditions (Liu et al 2012; Nunnari and Suomalainen 2012). In fact, recent transcriptomic studies of cultured skin fibroblasts from a large group of respiratory chain disorders, with various primary gene defects, revealed altered AMPK/FOXO3a/PPAR signalling, which could be reversed by treating the fibroblasts with nicotinic acid (a precursor for NAD^+^) to enhance sirtuin activity and improve cellular respiratory chain activity (Zhang et al 2013).

Although dysfunctional NAD^+^ signalling certainly may act to maintain and worsen the maladaptive stress responses, it does not explain how these signalling molecules become abnormal in the first place. Of course, for mitochondrial diseases with mutations in respiratory chain complexes, the decreased NAD^+^ could be a direct effect of a compromised respiratory chain activity, but for other IEM or diseases with more complex genetics, which do not directly affect respiratory chain function, other explanations may need to be put forward to explain their blunted repair responses. As discussed here, toxic misfolded proteins or accumulated substrates in IEM — but also glycation end products in diabetes (Picard et al 2014) and the formation of β-amyloids and tau aggregates in Alzheimer disease (Bobba et al 2013; Pizzimenti et al 2013) — may accumulate and interact with the respiratory chain and induce oxidative stress, and/or act as DAMPs to stimulate ROS production through inflammatory processes, as discussed above. If ROS persist, or is not properly controlled by the AMPK/Nrf2-linked repair pathways, the repair mechanisms may be overloaded with the increasing risk of irreversible damage to the respiratory chain and decline in mitochondrial respiration and signalling.

In fact, it has been suggested that during chronic oxidative stress, there is a bias towards redox deregulation by the introduction of irreversible structural damage, which may also hit repair enzymes. For example, it has been shown that intermolecular disulfide bond formation between heat shock factor 1 (HSF1) proteins causes trimerization and DNA binding to the proximal promoters of heat shock proteins, whereas an intramolecular disulfide bond formation inhibits the activity of the transcription factor (Calabrese et al 2012). Similarly the Lon protease can be inactivated by oxidants such as ONOO^−^ (Stanyer et al 2008). Therefore, we suggest that especially the level and duration of cellular ROS load drives the decline in cell repair mechanisms as exemplified by the studies on RR:MADD cells, discussed above.

It is increasingly established that the level of ROS itself may determine the type of the cell stress responses by modulating the activity of different redox-sensitive transcription factors, phosphatases and kinases. For instance, low ROS levels induce Nrf2 for the induction of several antioxidant and anti-inflammatory proteins as described above. Moderate increases in ROS can activate HIF-1α by oxidation of certain cysteine residues in HIF-1α regulatory proteins, whereas further oxidation of other residues at higher ROS levels inactivates HIF-1α and induces apoptosis (Page et al 2008; Wang et al 2012). In line with this, certain mutations in the ubiquinone-binding sites of complex I and complex II have been demonstrated to induce a pseudo-hypoxic metabolic switch from mitochondrial respiration towards glycolytic ATP production through activation of the Akt/HIF-1α pathway and down-regulation of activated AMPK in a ROS dose dependent manner (Guzy et al 2008; Sharma et al 2011).

**Fig. 4:**
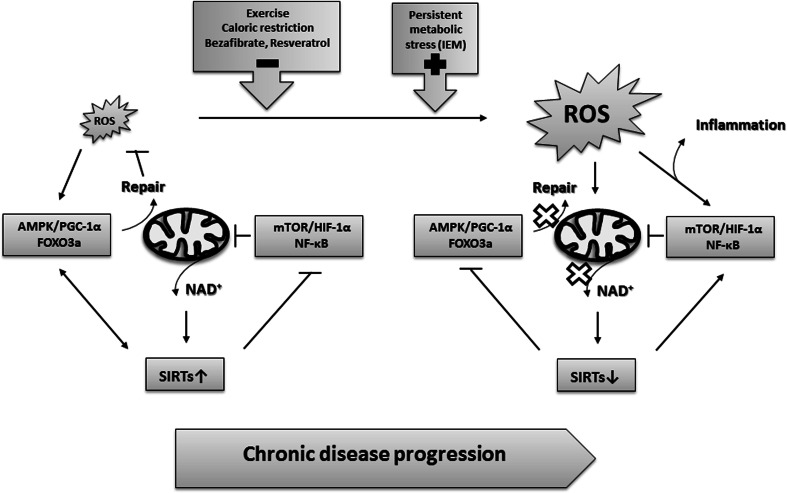
Molecular mechanisms of chronic disease progression in which persistent or increased ROS/damage load and/or decreased levels of NAD^+^ drive the transition from AMPK/PGC-1α/FOXO3a-linked repair responses towards pro-inflammatory responses controlled by the mTOR/HIF-1α/NF-κB axis. See text for further explanation

Thus, during chronic cell stress, lack of mitochondrial NAD^+^ to sustain activation of the AMPK/PGC-1α/FOXO3a axis, and/or activation of the mTOR/HIF-1α axis at increasing ROS levels may shift the cell from mitochondrial respiration and active repair responses towards a more glycolytic metabolism with compromised repair mechanisms. In both scenarios, ROS is mainly produced and propagated by a damaged respiratory chain (Fig. 4).

So what are the cellular or whole tissue/body advantages — if any — of this second line of adaptive stress responses, which seem to be widely conserved between different disease conditions? We have speculated that during persistent or increasing ROS load — during which the repair mechanisms may be overloaded and irreversible structural damage increasingly is introduced — the cell is forced to direct electrons away from a damaged respiratory chain membrane to prevent further ROS production that will otherwise induce membrane permeability transition pore opening, cytochrome c release and apoptotic cell death or necrotic tissue damage (Olsen et al 2013; Cornelius et al 2014b). The survival cost is impaired mitochondrial function, ROS signalling and repair mechanisms with decreased stress resistance and disturbed cell growth control. Alternatively, the decline in mitochondrial respiration and repair mechanisms is just an inappropriate consequence of stochastic damage to mitochondrial function and signalling. The precise molecular mechanisms and cellular consequences are still unknown, but certainly needed in order to know if targeting these pathways by mitochondrial enhancer drugs like bezafibrate and resveratrol will improve or worsen outcome as discussed below.

## Limitations and scientific/clinical applicability of the ROS triangle model

Based on recent studies we have here discussed how IEM, by production of pathological amounts of ROS, may induce chronic cell stress with non-resolving or persistent cell repair responses (first line of adaptive stress responses), or cause disturbed redox signalling with compromised cell repair responses (second line of adaptive stress) that make the cells more susceptible for stress induced cell death. We are aware that the proposed ROS triangle is a simplified model, not sufficiently comprehensive to explain the complexity of chronic disease development, and that many tissue samples from such diseases will show mixed cell stress responses as illustrated by the overlapping response curves in Fig. 3a. These antagonistic cell stress responses seem to be linked to distinct cellular metabolism through nutrient-sensing signalling growth pathways that control a Warburg-like shift from mitochondrial respiration towards cytosolic glycolysis. Some of the components — such as AMPK-linked induction of antioxidant enzymes, mitophagy and mitochondrial biogenesis — have been identified and characterized in some details. However, others — such as the regulation of the protein quality control and DNA repair systems, and the mTOR/HIF-1α mediated antagonistic cell stress responses — remain to be integrated to understand how to incorporate mitochondrial stress responses into understanding the altered nutrient-sensing signalling growth pathway that are increasingly revealed by system biological approaches.

Although we have argued that ROS is the most important triggering factor, which also drives the transition from one stage of the ROS triangle to another, also other mitochondrial signalling molecules like NAD^+^/NADH, AMP/ATP, acetyl-CoA, Ca^2+^, FAD etc. will change accordingly and add to maintain or progress the cellular stress responses (Nunnari and Suomalainen 2012; Guha and Avadhani 2013). Together with ROS, these mitochondrial signalling molecules allow rapid and specific post-translational regulation of stress proteins within hours of the stress triggering event and within days/weeks for transcriptional up-regulation of their expression. For longer lasting stress memory, epigenetic changes are involved. Metabolic reprogramming, and thereby stress responses, is determined epigentically (Wallace and Fan 2010; Gut and Verdin 2013). Therefore, the mechanisms by which cells cope with stress, through adaptive responses to induce homeostasis and survive, is much more complex than proposed by the ROS triangle model. To move the model forward towards clinical applicability it is important to investigate in more detail the redox and bioenergetics signalling pathways that drive cellular stress responses and chronic disease development, and investigate the cellular consequences of targeting them by antioxidant and mitochondrial enhancement treatment.

As discussed above, boosting mitochondrial biogenesis using pharmacological approaches such as bezafibrate, resveratrol and AICAR have been tried in cells from patients with IEM. Altogether, these ex vivo studies showed increases in FAO and/or OXPHOS capacity and clearly suggest a therapeutic potential of the drugs for ameliorating the cellular energy deficiency in patients. None of the studies have investigated the effect of the treatments on oxidative and inflammatory stress and cell survival, which may well play an additional role in at least some patients. We have suggested that down-regulation of mitochondrial respiration, to decrease the source of ROS, is a cell survival mechanism to compensate for redox stress and an overloaded repair system and prevent cell death. Decreasing ROS production by reducing complex I activity has been shown to be an essential element in the mitochondrial reprograming induced by HIF-1α (Tello et al 2011). In this respect the benefit of mitochondrial enhancement treatment, may be better in the early phase of disease progression than in later stages, where irreversible oxidative damage to the respiratory chain has already occurred and treatment may even be harmful. Because of the IEM, such artificial and persistent induction of mitochondrial biogenesis theoretically may increase the risk of molecular damage spreading into the mitochondrial network with the risk of promoting further ROS production, pro-inflammation and/or increased cell death in the long term. Accordingly, clinical effect of bezafibrate treatment in patients with long-chain fatty acid oxidation disorders (Bonnefont et al 2009, 2010; Bastin et al 2014) have been questioned based on a recent study, where therapeutic effects were tested during exercise cycle tests (Orngreen et al 2014, 2015). It is likely that toxic levels of ROS — induced by the combined exercise and bezafibrate treatment — may have overwhelmed the repair mechanisms and shifted cell stress adaptation towards pro-inflammatory/death mechanisms. Alternatively, patient cells showing beneficial effects may have milder gene defects or be at the early stage of disease progression, or in a metabolic condition, where activation of mitochondrial biogenesis and repair mechanisms will allow the production and selection of a more healthy pool of mitochondria, providing more energy for coping with and adapting to the gene defects. Similar, variability has been reported in the biochemical and physiological outcome of bezafibrate treatment in mouse models of primary OXPHOS diseases (Komen and Thorburn 2014), reflecting that translation of ex vivo studies of cells in culture to in vivo clinical outcomes are not straightforward. Studies that test the long-term consequences of mitochondrial enhancement therapy and monitor metabolic enhancement in relation to oxidative stress and mitochondrial stress responses, and in relation to severity (mild versus severe gene defects) and duration (young versus older patients) of disease phenotype are therefore highly needed. Also, in those conditions, where ROS are a major concern, it will be important to develop or test therapy that can inhibit ROS production without interfering with redox regulation of endogenous repair mechanisms, and combine such ROS scavenging therapy with mitochondrial enhancement therapy. Bezafibrate is a dual activator of PPARα and PPARβ, the latter one being responsible for improvement in FAO capacity (Bastin 2014). Even though both of these PPARs have been implicated in alleviating oxidative stress, the exact molecular mechanisms remain to be elucidated, and their antioxidant properties seem to depend on various metabolic and pathological conditions (Kim and Yang 2013). In this respect, it may be better to treat with resveratrol. Resveratrol activates mitochondrial biogenesis at AMPK by boosting NAD^+^ and SIRT activities, and will activate not only mitochondrial biogenesis but also the antioxidant system and mitophagy to protect the cells from ROS produced during mitochondrial biogenesis (Fig. 2). Aerobic exercise is another combined mitochondrial antioxidant and enhancement therapy. Exercise has been shown to increase muscle performance in patients with mitochondrial myopathies while partly reverting oxidative stress (Siciliano et al 2012). Finally, CoQ10 has combined ROS scavenger and mitochondrial enhancement function (Lee et al 2012), and it has shown beneficial effects in some IEM, like MERRF (De la Mata et al 2012). However, in other diseases, including RR:MADD, CoQ10 has had only minor effects (Cornelius et al 2013, 2014b). In later disease stages, or during intensive ROS load, the lipid membranes may be too damaged for CoQ10 or other ROS scavengers to have beneficial effects, and lipid replacement therapy could be tried (Nicolson and Ash 2014). In accordance with this theory, MERRF cells, which show a good response to CoQ10 treatment, are in the early phase of chronic disease progression with activation of “first line of adaptive stress responses”. RR:MADD cells, which show limiting response to CoQ10 treatment, seem to be at a later state of chronic disease progression with activation of mostly “second line of adaptive stress responses”. These reflections underscore the importance of elucidating the precise mechanisms by which ROS is produced and propagated in different IEM. Another challenge for clinical applicability of the ROS triangle model is that because of the metabolic nature of the stress responses, different stress responses may exist in different tissues (Zhang et al 2013) and even coexist in the same tissue; ex immune cells (Liu et al 2012).

The establishment of neonatal screening programs for a number of IEM has enabled early or pre-symptomatic identification of patients and their causative gene defects. In as much as oxidative stress and mitochondrial dysfunction may be an important factor in some of the patient’s disease development, new approaches and methods, such as blood biomarkers and non-invasive imaging techniques, for clinical evaluation of mitochondrial function and stress responses, are needed for pre-symptomatic prognosis and treatment decisions. Although still in its infancy, the ROS triangle model may present a framework to move forward with new ideas for the design of appropriate experiments to monitor stress responses and activated nutrient signalling pathways during disease progression and in response to therapy and disease triggering factors.

## Outlook

**Fig. 5:**
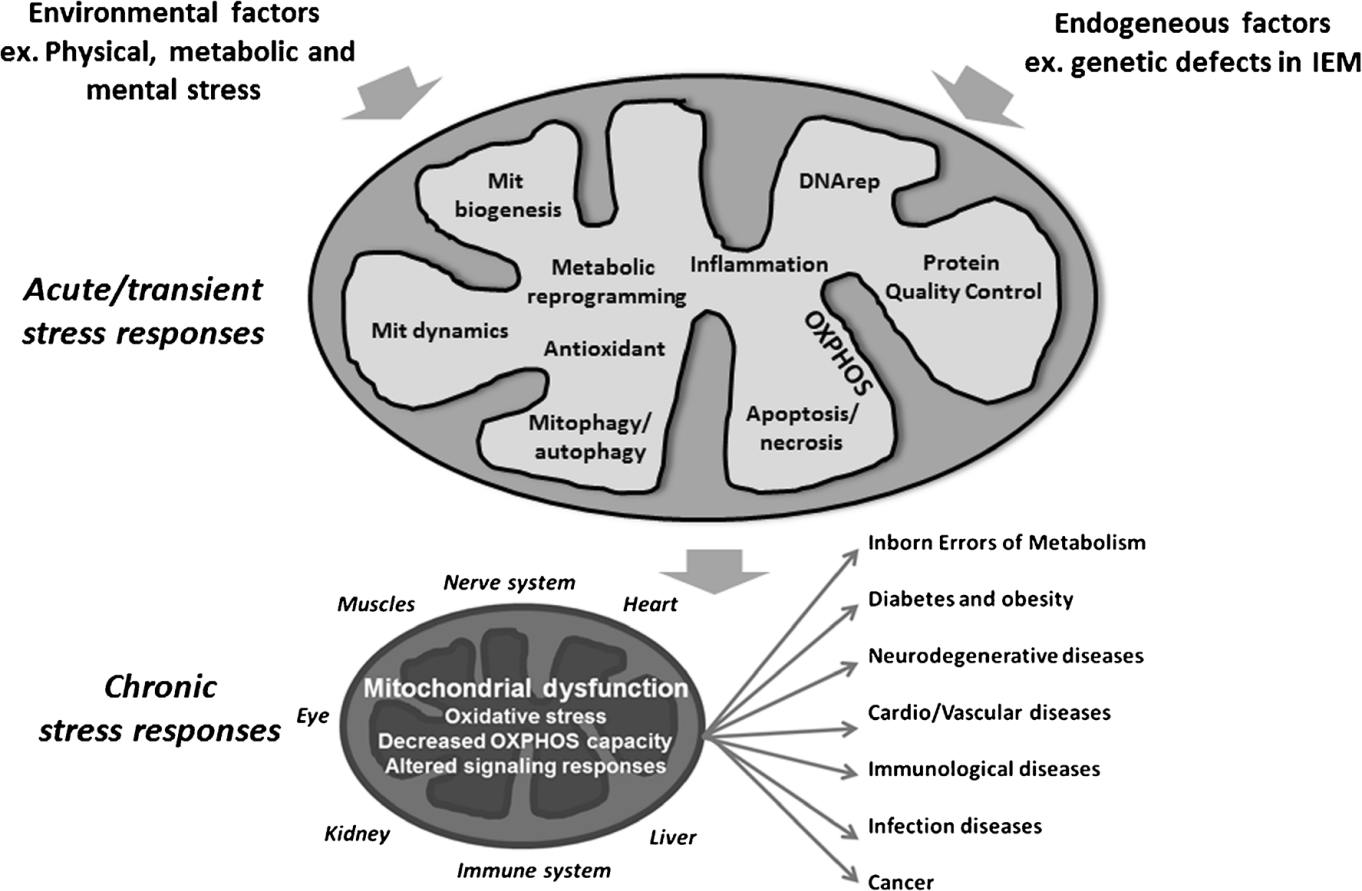
Mitochondria are present in most tissues, and decline in mitochondrial function and signalling is a common finding in many chronic diseases and ageing. As such, we suggest that comorbidity is clinical expression of mitochondrial dysfunction, and that research in chronic disease development and prevention should be directed towards targeting mitochondrial signalling and pathways

A recent special issue of Science celebrated a resumed interest in metabolism and discussed a new metabolic paradigm for disease (Ray 2010). The increasing use of system biological approaches has driven this new understanding for disease development with a realization that altered nutrient-sensing signalling growth pathways and metabolic reprogramming, towards a more glycolytic metabolism, drives the initiation or progression of most diseases, like cancer, diabetes, neurodegenerative diseases, cardiovascular diseases and IEM. As discussed in the present review, mitochondria are absolutely central for this metabolic paradigm for disease as these organelles do not only provide the cell with energy and biosynthetic intermediates for cell growth, but also for support of ROS- and energy-dependent mechanisms like those involved in cell defense and the repair of cell and tissue damage (Fig. 5). Therefore, to move present knowledge towards clinical applicability will require scientists to incorporate mitochondrial stress responses and the principle of the ROS triangle model into understanding the altered nutrient-sensing signalling growth pathway and metabolic reprogramming revealed in many chronic diseases during the last decade.

## References

[CR1] Ahuja P, Wanagat J, Wang Z (2013). Divergent mitochondrial biogenesis responses in human cardiomyopathy. Circulation.

[CR2] Aires V, Delmas D, Le Bachelier C (2014). Stilbenes and resveratrol metabolites improve mitochondrial fatty acid oxidation defects in human fibroblasts. Orphanet J Rare Dis.

[CR3] Aquilano K, Baldelli S, Pagliei B, Cannata SM, Rotilio G, Ciriolo MR (2013). p53 orchestrates the PGC-1alpha-mediated antioxidant response upon mild redox and metabolic imbalance. Antioxid Redox Signal.

[CR4] Archer SL (2013). Mitochondrial dynamics–mitochondrial fission and fusion in human diseases. N Engl J Med.

[CR5] Baldelli S, Aquilano K, Ciriolo MR (2013). Punctum on two different transcription factors regulated by PGC-1alpha: nuclear factor erythroid-derived 2-like 2 and nuclear respiratory factor 2. Biochim Biophys Acta.

[CR6] Baldelli S, Aquilano K, Ciriolo MR (2014). PGC-1alpha buffers ROS-mediated removal of mitochondria during myogenesis. Cell Death Dis.

[CR7] Bastin J (2014). Regulation of mitochondrial fatty acid β-oxidation in human: what can we learn from inborn fatty acid β-oxidation deficiencies?. Biochimie.

[CR8] Bastin J, Bonnefont JP, Djouadi F, Bresson JL (2014) Should the beneficial impact of bezafibrate on fatty acid oxidation disorders be questioned? J Inherit Metab Dis 10.1007/s10545-014-9775-7 25310995

[CR9] Bates MG, Bourke JP, Giordano C, d’Amati G, Turnbull DM, Taylor RW (2012). Cardiac involvement in mitochondrial DNA disease: clinical spectrum, diagnosis, and management. Eur Heart J.

[CR10] Bauer S, Wanninger J, Neumeier M (2011). Elevated free fatty acids and impaired adiponectin bioactivity contribute to reduced SOD2 protein in monocytes of type 2 diabetes patients. Exp Mol Pathol.

[CR11] Bell EL, Emerling BM, Ricoult SJ, Guarente L (2011). SirT3 suppresses hypoxia inducible factor 1alpha and tumor growth by inhibiting mitochondrial ROS production. Oncogene.

[CR12] Bobba A, Amadoro G, Valenti D, Corsetti V, Lassandro R, Atlante A (2013). Mitochondrial respiratory chain complexes I and IV are impaired by beta-amyloid via direct interaction and through complex I-dependent ROS production, respectively. Mitochondrion.

[CR13] Bolisetty S, Jaimes EA (2013). Mitochondria and reactive oxygen species: physiology and pathophysiology. Int J Mol Sci.

[CR14] Bonnefont JP, Bastin J, Behin A, Djouadi F (2009). Bezafibrate for an inborn mitochondrial beta-oxidation defect. N Engl J Med.

[CR15] Bonnefont JP, Bastin J, Laforet P (2010). Long-term follow-up of bezafibrate treatment in patients with the myopathic form of carnitine palmitoyltransferase 2 deficiency. Clin Pharmacol Ther.

[CR16] Brenmoehl J, Hoeflich A (2013). Dual control of mitochondrial biogenesis by sirtuin 1 and sirtuin 3. Mitochondrion.

[CR17] Calabrese V, Cornelius C, Dinkova-Kostova AT (2012). Cellular stress responses, hormetic phytochemicals and vitagenes in aging and longevity. Biochim Biophys Acta.

[CR18] Casarin A, Giorgi G, Pertegato V (2012). Copper and bezafibrate cooperate to rescue cytochrome c oxidase deficiency in cells of patients with SCO2 mutations. Orphanet J Rare Dis.

[CR19] Charniot JC, Sutton A, Bonnefont-Rousselot D (2011). Manganese superoxide dismutase dimorphism relationship with severity and prognosis in cardiogenic shock due to dilated cardiomyopathy. Free Radic Res.

[CR20] Chiribau CB, Cheng L, Cucoranu IC, Yu YS, Clempus RE, Sorescu D (2008). FOXO3A regulates peroxiredoxin III expression in human cardiac fibroblasts. J Biol Chem.

[CR21] Cornelius N, Frerman FE, Corydon TJ (2012). Molecular mechanisms of riboflavin responsiveness in patients with ETF-QO variations and multiple acyl-CoA dehydrogenation deficiency. Hum Mol Genet.

[CR22] Cornelius N, Byron C, Hargreaves I (2013). Secondary coenzyme Q10 deficiency and oxidative stress in cultured fibroblasts from patients with riboflavin responsive multiple Acyl-CoA dehydrogenation deficiency. Hum Mol Genet.

[CR23] Cornelius N, Gregersen N, Tümer Z, Olsen RK (2014a) Oxidative stress and mitochondrial defect in neuromuscular disorders: source or symptoms? In: Oxidative stress: causes, role in diseases and biological effects. Nova Biomedical, Huntington

[CR24] Cornelius N, Corydon TJ, Gregersen N, Olsen RK (2014). Cellular consequences of oxidative stress in riboflavin responsive multiple acyl-CoA dehydrogenation deficiency patient fibroblasts. Hum Mol Genet.

[CR25] Dai DF, Chen T, Wanagat J (2010). Age-dependent cardiomyopathy in mitochondrial mutator mice is attenuated by overexpression of catalase targeted to mitochondria. Aging Cell.

[CR26] De la Mata M, Garrido-Maraver J, Cotan D (2012). Recovery of MERRF fibroblasts and cybrids pathophysiology by coenzyme Q10. Neurotherapeutics.

[CR27] De Paepe B, Vandemeulebroecke K, Smet J (2014). Effect of resveratrol on cultured skin fibroblasts from patients with oxidative phosphorylation defects. Phytother Res.

[CR28] Demirovic D, Rattan SI (2013). Establishing cellular stress response profiles as biomarkers of homeodynamics, health and hormesis. Exp Gerontol.

[CR29] Desquiret-Dumas V, Gueguen N, Leman G (2013). Resveratrol induces a mitochondrial complex I-dependent increase in NADH oxidation responsible for sirtuin activation in liver cells. J Biol Chem.

[CR30] Djouadi F, Aubey F, Schlemmer D (2005). Bezafibrate increases very-long-chain acyl-CoA dehydrogenase protein and mRNA expression in deficient fibroblasts and is a potential therapy for fatty acid oxidation disorders. Hum Mol Genet.

[CR31] Dröse S, Brandt U (2012). Molecular mechanisms of superoxide production by the mitochondrial respiratory chain. Adv Exp Med Biol.

[CR32] Elstner M, Turnbull DM (2012). Transcriptome analysis in mitochondrial disorders. Brain Res Bull.

[CR33] Figge MT, Osiewacz HD, Reichert AS (2013). Quality control of mitochondria during aging: is there a good and a bad side of mitochondrial dynamics?. Bioessays.

[CR34] Finley LW, Carracedo A, Lee J (2011). SIRT3 opposes reprogramming of cancer cell metabolism through HIF1alpha destabilization. Cancer Cell.

[CR35] Fischer F, Hamann A, Osiewacz HD (2012). Mitochondrial quality control: an integrated network of pathways. Trends Biochem Sci.

[CR36] Gempel K, Topaloglu H, Talim B (2007). The myopathic form of coenzyme Q10 deficiency is caused by mutations in the electron-transferring-flavoprotein dehydrogenase (ETFDH) gene. Brain.

[CR37] Gobin-Limballe S, Djouadi F, Aubey F (2007). Genetic basis for correction of very-long-chain acyl-coenzyme A dehydrogenase deficiency by bezafibrate in patient fibroblasts: toward a genotype-based therapy. Am J Hum Genet.

[CR38] Golubitzky A, Dan P, Weissman S (2011). Screening for active small molecules in mitochondrial complex I deficient patient’s fibroblasts, reveals AICAR as the most beneficial compound. PLoS One.

[CR39] Goncalves RL, Quinlan CL, Perevoshchikova IV (2015). Sites of superoxide and hydrogen peroxide production by muscle mitochondria assessed ex vivo under conditions mimicking rest and exercise. J Biol Chem.

[CR40] Goodman SI, Binard RJ, Woontner MR, Frerman FE (2002). Glutaric acidemia type II: gene structure and mutations of the electron transfer flavoprotein:ubiquinone oxidoreductase (ETF:QO) gene. Mol Genet Metab.

[CR41] Grant CM (2008). Metabolic reconfiguration is a regulated response to oxidative stress. J Biol.

[CR42] Gregersen N, Bross P (2010). Protein misfolding and cellular stress: an overview. Methods Mol Biol.

[CR43] Gregersen N, Andresen BS, Pedersen CB, Olsen RK, Corydon TJ, Bross P (2008). Mitochondrial fatty acid oxidation defects–remaining challenges. J Inherit Metab Dis.

[CR44] Grunert SC (2014). Clinical and genetical heterogeneity of late-onset multiple acyl-coenzyme A dehydrogenase deficiency. Orphanet J Rare Dis.

[CR45] Guha M, Avadhani NG (2013). Mitochondrial retrograde signaling at the crossroads of tumor bioenergetics, genetics and epigenetics. Mitochondrion.

[CR46] Guo J, Lemire BD (2003). The ubiquinone-binding site of the saccharomyces cerevisiae succinate-ubiquinone oxidoreductase is a source of superoxide. J Biol Chem.

[CR47] Gut P, Verdin E (2013). The nexus of chromatin regulation and intermediary metabolism. Nature.

[CR48] Guzy RD, Sharma B, Bell E, Chandel NS, Schumacker PT (2008). Loss of the SdhB, but Not the SdhA, subunit of complex II triggers reactive oxygen species-dependent hypoxia-inducible factor activation and tumorigenesis. Mol Cell Biol.

[CR49] Hagenbuchner J, Ausserlechner MJ (2013). Mitochondria and FOXO3: breath or die. Front Physiol.

[CR50] Hayashi G, Shen Y, Pedersen TL, Newman JW, Pook M, Cortopassi G (2014). Frataxin deficiency increases cyclooxygenase 2 and prostaglandins in cell and animal models of Friedreich’s ataxia. Hum Mol Genet.

[CR51] Heiss EH, Schachner D, Zimmermann K, Dirsch VM (2013). Glucose availability is a decisive factor for Nrf2-mediated gene expression. Redox Biol.

[CR52] Higdon A, Diers AR, Oh JY, Landar A, Darley-Usmar VM (2012). Cell signalling by reactive lipid species: new concepts and molecular mechanisms. Biochem J.

[CR53] Holley AK, Dhar SK, St Clair DK (2013). Curbing cancer’s sweet tooth: is there a role for MnSOD in regulation of the Warburg effect?. Mitochondrion.

[CR54] Houtkooper RH, Pirinen E, Auwerx J (2012). Sirtuins as regulators of metabolism and healthspan. Nat Rev Mol Cell Biol.

[CR55] Huang J, Lemire BD (2009). Mutations in the C. elegans succinate dehydrogenase iron-sulfur subunit promote superoxide generation and premature aging. J Mol Biol.

[CR56] Huang D, Li T, Li X (2014). HIF-1-mediated suppression of acyl-CoA dehydrogenases and fatty acid oxidation is critical for cancer progression. Cell Rep.

[CR57] Jain A, Lamark T, Sjottem E (2010). p62/SQSTM1 is a target gene for transcription factor NRF2 and creates a positive feedback loop by inducing antioxidant response element-driven gene transcription. J Biol Chem.

[CR58] Kansanen E, Jyrkkanen HK, Levonen AL (2012). Activation of stress signaling pathways by electrophilic oxidized and nitrated lipids. Free Radic Biol Med.

[CR59] Kauppinen A, Suuronen T, Ojala J et al (2013) Antagonistic crosstalk between NF-kB and SIRT1 in the regulation of inflammation and metabolic disorders. Cell Sigal 25:1939–1948 10.1016/j.cellsig.2013.06.007 23770291

[CR60] Kemper MF, Stirone C, Krause DN, Duckles SP, Procaccio V (2014). Genomic and non-genomic regulation of PGC1 isoforms by estrogen to increase cerebral vascular mitochondrial biogenesis and reactive oxygen species protection. Eur J Pharmacol.

[CR61] Kim T, Yang Q (2013). Peroxisome-proliferator-activated receptors regulate redox signaling in the cardiovascular system. World J Cardiol.

[CR62] Kolker S, Burgard P, Sauer SW, Okun JG (2013). Current concepts in organic acidurias: understanding intra- and extracerebral disease manifestation. J Inherit Metab Dis.

[CR63] Komatsu M, Kurokawa H, Waguri S (2010). The selective autophagy substrate p62 activates the stress responsive transcription factor Nrf2 through inactivation of Keap1. Nat Cell Biol.

[CR64] Komen JC, Thorburn DR (2014). Turn up the power - pharmacological activation of mitochondrial biogenesis in mouse models. Br J Pharmacol.

[CR65] Koopman WJ, Willems PH, Smeitink JA (2012). Monogenic mitochondrial disorders. N Engl J Med.

[CR66] Koopman WJ, Distelmaier F, Smeitink JA, Willems PH (2013). OXPHOS mutations and neurodegeneration. EMBO J.

[CR67] Kops GJ, Dansen TB, Polderman PE (2002). Forkhead transcription factor FOXO3a protects quiescent cells from oxidative stress. Nature.

[CR68] Kotiadis VN, Duchen MR, Osellame LD (2014). Mitochondrial quality control and communications with the nucleus are important in maintaining mitochondrial function and cell health. Biochim Biophys Acta.

[CR69] Kwak MK, Wakabayashi N, Greenlaw JL, Yamamoto M, Kensler TW (2003). Antioxidants enhance mammalian proteasome expression through the Keap1-Nrf2 signaling pathway. Mol Cell Biol.

[CR70] Laplante M, Sabatini DM (2012). mTOR signaling in growth control and disease. Cell.

[CR71] Lee SK, Lee JO, Kim JH (2012). Coenzyme Q10 increases the fatty acid oxidation through AMPK-mediated PPARalpha induction in 3T3-L1 preadipocytes. Cell Signal.

[CR72] Levonen AL, Hill BG, Kansanen E, Zhang J, Darley-Usmar VM (2014). Redox regulation of antioxidants, autophagy, and the response to stress: implications for electrophile therapeutics. Free Radic Biol Med.

[CR73] Li XN, Song J, Zhang L (2009). Activation of the AMPK-FOXO3 pathway reduces fatty acid-induced increase in intracellular reactive oxygen species by upregulating thioredoxin. Diabetes.

[CR74] Li H, Fukuda S, Hasegawa Y (2010). Effect of heat stress and bezafibrate on mitochondrial beta-oxidation: comparison between cultured cells from normal and mitochondrial fatty acid oxidation disorder children using in vitro probe acylcarnitine profiling assay. Brain Dev.

[CR75] Lim JH, Lee YM, Chun YS, Chen J, Kim JE, Park JW (2010). Sirtuin 1 modulates cellular responses to hypoxia by deacetylating hypoxia-inducible factor 1alpha. Mol Cell.

[CR76] Liu TF, Brown CM, El GM (2012). Fueling the flame: bioenergy couples metabolism and inflammation. J Leukoc Biol.

[CR77] Lopes CA, Le BC, Mathieu L (2014). Beneficial effects of resveratrol on respiratory chain defects in patients’ fibroblasts involve estrogen receptor and estrogen-related receptor alpha signaling. Hum Mol Genet.

[CR78] Lopez-Lluch G, Hunt N, Jones B (2006). Calorie restriction induces mitochondrial biogenesis and bioenergetic efficiency. Proc Natl Acad Sci U S A.

[CR79] Marinho HS, Real C, Cyrne L, Soares H, Antunes F (2014). Hydrogen peroxide sensing, signaling and regulation of transcription factors. Redox Biol.

[CR80] Marmolino D, Manto M, Acquaviva F (2010). PGC-1alpha down-regulation affects the antioxidant response in Friedreich’s ataxia. PLoS One.

[CR81] Milisav I, Poljsak B, Suput D (2012). Adaptive response, evidence of cross-resistance and its potential clinical use. Int J Mol Sci.

[CR82] Mohelnikova-Duchonova B, Marsakova L, Vrana D (2011). Superoxide dismutase and nicotinamide adenine dinucleotide phosphate: quinone oxidoreductase polymorphisms and pancreatic cancer risk. Pancreas.

[CR83] Ngo JK, Pomatto LC, Davies KJ (2013). Upregulation of the mitochondrial Lon Protease allows adaptation to acute oxidative stress but dysregulation is associated with chronic stress, disease, and aging. Redox Biol.

[CR84] Nicolson GL, Ash ME (2014). Lipid replacement therapy: a natural medicine approach to replacing damaged lipids in cellular membranes and organelles and restoring function. Biochim Biophys Acta.

[CR85] Nunnari J, Suomalainen A (2012). Mitochondria: in sickness and in health. Cell.

[CR86] Olsen RK, Andresen BS, Christensen E, Bross P, Skovby F, Gregersen N (2003). Clear relationship between ETF/ETFDH genotype and phenotype in patients with multiple acyl-CoA dehydrogenation deficiency. Hum Mutat.

[CR87] Olsen RK, Olpin SE, Andresen BS (2007). ETFDH mutations as a major cause of riboflavin-responsive multiple acyl-CoA dehydrogenation deficiency. Brain.

[CR88] Olsen RK, Cornelius N, Gregersen N (2013). Genetic and cellular modifiers of oxidative stress: what can we learn from fatty acid oxidation defects?. Mol Genet Metab.

[CR89] Olsson J, Jacobson TA, Paulsson JM (2011). Expression of neutrophil SOD2 is reduced after lipopolysaccharide stimulation: a potential cause of neutrophil dysfunction in chronic kidney disease. Nephrol Dial Transplant.

[CR90] Orngreen MC, Madsen KL, Preisler N (2014). Bezafibrate in skeletal muscle fatty acid oxidation disorders: a randomized clinical trial. Neurology.

[CR91] Orngreen MC, Vissing J, Laforet P (2015) No effect of bezafibrate in patients with CPTII and VLCAD deficiencies. J Inherit Metab Dis 10.1007/s10545-014-9779-3 25331908

[CR92] Page EL, Chan DA, Giaccia AJ, Levine M, Richard DE (2008). Hypoxia-inducible factor-1alpha stabilization in nonhypoxic conditions: role of oxidation and intracellular ascorbate depletion. Mol Biol Cell.

[CR93] Palacios OM, Carmona JJ, Michan S (2009). Diet and exercise signals regulate SIRT3 and activate AMPK and PGC-1alpha in skeletal muscle. Aging (Albany NY).

[CR94] Patsi J, Kervinen M, Finel M, Hassinen IE (2008). Leber hereditary optic neuropathy mutations in the ND6 subunit of mitochondrial complex I affect ubiquinone reduction kinetics in a bacterial model of the enzyme. Biochem J.

[CR95] Piantadosi CA, Suliman HB (2012). Redox regulation of mitochondrial biogenesis. Free Radic Biol Med.

[CR96] Picard M, Juster RP, McEwen BS (2014). Mitochondrial allostatic load puts the ‘gluc’ back in glucocorticoids. Nat Rev Endocrinol.

[CR97] Pinti M, Gibellini L, De BS (2011). Functional characterization of the promoter of the human Lon protease gene. Mitochondrion.

[CR98] Pizzimenti S, Ciamporcero E, Daga M (2013). Interaction of aldehydes derived from lipid peroxidation and membrane proteins. Front Physiol.

[CR99] Polvani S, Tarocchi M, Galli A (2012). PPARgamma and oxidative stress: Con(beta) catenating NRF2 and FOXO. PPAR Res.

[CR100] Ray LB (2010). Metabolism. Metabolism is not boring. Introduction. Science.

[CR101] Ristow M, Schmeisser K (2014). Mitohormesis: promoting health and lifespan by increased levels of reactive oxygen species (ROS). Dose-Response.

[CR102] Rocha H, Ferreira R, Carvalho J (2011). Characterization of mitochondrial proteome in a severe case of ETF-QO deficiency. J Proteomics.

[CR103] Rodell A, Rasmussen LJ, Bergersen LH, Singh KK, Gjedde A (2013). Natural selection of mitochondria during somatic lifetime promotes healthy aging. Front Neuroenerg.

[CR104] Rodrigues JV, Gomes CM (2012). Mechanism of superoxide and hydrogen peroxide generation by human electron-transfer flavoprotein and pathological variants. Free Radic Biol Med.

[CR105] Safdar A, Little JP, Stokl AJ, Hettinga BP, Akhtar M, Tarnopolsky MA (2011). Exercise increases mitochondrial PGC-1alpha content and promotes nuclear-mitochondrial cross-talk to coordinate mitochondrial biogenesis. J Biol Chem.

[CR106] Salminen A, Kaarniranta K (2009). Regulation of the aging process by autophagy. Trends Mol Med.

[CR107] Salminen A, Hyttinen JM, Kaarniranta K (2011). AMP-activated protein kinase inhibits NF-kappaB signaling and inflammation: impact on healthspan and lifespan. J Mol Med (Berl).

[CR108] Sanchis-Gomar F, Garcia-Gimenez JL, Gomez-Cabrera MC, Pallardo FV (2014). Mitochondrial biogenesis in health and disease. Molecular and therapeutic approaches. Curr Pharm Des.

[CR109] Scarpulla RC, Vega RB, Kelly DP (2012). Transcriptional integration of mitochondrial biogenesis. Trends Endocrinol Metab.

[CR110] Schapira AH (2006). Mitochondrial disease. Lancet.

[CR111] Schumacker PT (2011). SIRT3 controls cancer metabolic reprogramming by regulating ROS and HIF. Cancer Cell.

[CR112] Scott JL, Gabrielides C, Davidson RK (2010). Superoxide dismutase downregulation in osteoarthritis progression and end-stage disease. Ann Rheum Dis.

[CR113] Sebastiani M, Giordano C, Nediani C (2007). Induction of mitochondrial biogenesis is a maladaptive mechanism in mitochondrial cardiomyopathies. J Am Coll Cardiol.

[CR114] Sharma LK, Fang H, Liu J, Vartak R, Deng J, Bai Y (2011). Mitochondrial respiratory complex I dysfunction promotes tumorigenesis through ROS alteration and AKT activation. Hum Mol Genet.

[CR115] Shirwany NA, Zou MH (2014). AMPK: a cellular metabolic and redox sensor. A minireview. Front Biosci (Landmark Ed).

[CR116] Siciliano G, Simoncini C, Lo Gerfo A (2012). Effects of aerobic training on exercise-related oxidative stress in mitochondrial myopathies. Neuromuscul Disord.

[CR117] Smeitink J, van den Heuvel L, DiMauro S (2001). The genetics and pathology of oxidative phosphorylation. Nat Rev Genet.

[CR118] Song Y, Selak MA, Watson CT (2009). Mechanisms underlying metabolic and neural defects in zebrafish and human multiple acyl-CoA dehydrogenase deficiency (MADD). PLoS One.

[CR119] Stanyer L, Jorgensen W, Hori O, Clark JB, Heales SJ (2008). Inactivation of brain mitochondrial Lon protease by peroxynitrite precedes electron transport chain dysfunction. Neurochem Int.

[CR120] St-Pierre J, Buckingham JA, Roebuck SJ (2002). Topology of superoxide production from different sites in the mitochondrial electron transport chain. J Biol Chem.

[CR121] Suliman HB, Piantadosi CA (2014). Mitochondrial biogenesis: regulation by endogenous gases during inflammation and organ stress. Curr Pharm Des.

[CR122] Sundaresan NR, Gupta M, Kim G, Rajamohan SB, Isbatan A, Gupta MP (2009). Sirt3 blocks the cardiac hypertrophic response by augmenting Foxo3a-dependent antioxidant defense mechanisms in mice. J Clin Invest.

[CR123] Tabas I, Glass CK (2013). Anti-inflammatory therapy in chronic disease: challenges and opportunities. Science.

[CR124] Tello D, Balsa E, Acosta-Iborra B (2011). Induction of the mitochondrial NDUFA4L2 protein by HIF-1α decreases oxygen consumption by inhibiting complex I activity. Cell Metab.

[CR125] Vander Heiden MG, Cantley LC, Thompson CB (2009). Understanding the Warburg effect: the metabolic requirements of cell proliferation. Science.

[CR126] Vives-Bauza C, Zhou C, Huang Y (2010). PINK1-dependent recruitment of Parkin to mitochondria in mitophagy. Proc Natl Acad Sci U S A.

[CR127] Wajner M, Goodman SI (2011). Disruption of mitochondrial homeostasis in organic acidurias: insights from human and animal studies. J Bioenerg Biomembr.

[CR128] Wallace DC, Fan W (2010). Energetics, epigenetics, mitochondrial genetics. Mitochondrion.

[CR129] Wang Y, Yang J, Yang K (2012). The biphasic redox sensing of SENP3 accounts for the HIF-1 transcriptional activity shift by oxidative stress. Acta Pharmacol Sin.

[CR130] Watmough NJ, Frerman FE (2010). The electron transfer flavoprotein: ubiquinone oxidoreductases. Biochim Biophys Acta.

[CR131] Wei YH, Wu SB, Ma YS, Lee HC (2009). Respiratory function decline and DNA mutation in mitochondria, oxidative stress and altered gene expression during aging. Chang Gung Med J.

[CR132] Wen B, Dai T, Li W (2010). Riboflavin-responsive lipid-storage myopathy caused by ETFDH gene mutations. J Neurol Neurosurg Psychiatry.

[CR133] Wu SB, Wei YH (2012). AMPK-mediated increase of glycolysis as an adaptive response to oxidative stress in human cells: implication of the cell survival in mitochondrial diseases. Biochim Biophys Acta.

[CR134] Wu SB, Ma YS, Wu YT, Chen YC, Wei YH (2010). Mitochondrial DNA mutation-elicited oxidative stress, oxidative damage, and altered gene expression in cultured cells of patients with MERRF syndrome. Mol Neurobiol.

[CR135] Wu SB, Wu YT, Wu TP, Wei YH (2014). Role of AMPK-mediated adaptive responses in human cells with mitochondrial dysfunction to oxidative stress. Biochim Biophys Acta.

[CR136] Yamaguchi S, Li H, Purevsuren J (2012). Bezafibrate can be a new treatment option for mitochondrial fatty acid oxidation disorders: evaluation by in vitro probe acylcarnitine assay. Mol Genet Metab.

[CR137] Yechoor VK, Patti ME, Ueki K (2004). Distinct pathways of insulin-regulated versus diabetes-regulated gene expression: an in vivo analysis in MIRKO mice. Proc Natl Acad Sci U S A.

[CR138] Youle RJ, van der Bliek AM (2012). Mitochondrial fission, fusion, and stress. Science.

[CR139] Zhang Q, Raoof M, Chen Y (2010). Circulating mitochondrial DAMPs cause inflammatory responses to injury. Nature.

[CR140] Zhang Z, Tsukikawa M, Peng M (2013). Primary respiratory chain disease causes tissue-specific dysregulation of the global transcriptome and nutrient-sensing signaling network. PLoS One.

[CR141] Zhou R, Yazdi AS, Menu P, Tschopp J (2011). A role for mitochondria in NLRP3 inflammasome activation. Nature.

[CR142] Zolkipli Z, Pedersen CB, Lamhonwah AM, Gregersen N, Tein I (2011). Vulnerability to oxidative stress in vitro in pathophysiology of mitochondrial short-chain acyl-CoA dehydrogenase deficiency: response to antioxidants. PLoS One.

